# Integrating Traditional Machine Learning and Convolutional Neural Networks Into the Geometric Morphometric Pipeline for Taxonomic Classification (GM-ML-CNN)

**DOI:** 10.1093/iob/obag048

**Published:** 2026-08-21

**Authors:** G Deku, R Combey

**Affiliations:** Ministry of Health, Health Training Institution Secretariat, Accra School of Hygiene, Environmental Health, Accra, Greater Accra 0000, Ghana; Department of Conservation Biology and Entomology, School of Biological Science; University of Cape Coast, Cape coast, Central 0000, Ghana; Department of Conservation Biology and Entomology, School of Biological Science; University of Cape Coast, Cape coast, Central 0000, Ghana

## Abstract

Geometric morphometrics (GM) is a well-established and cost-effective approach for quantifying shape variation and has been widely applied over the past few decades to resolve morphological differentiation and to discriminate among closely related taxa. A key analytical advantage of GM lies in its structured workflow, which transforms complex morphological features into multivariate shape data while inherently managing dimensionality. Consequently, GM outputs serve as powerful, optimized feature sets for training machine learning classifiers in taxonomic and ecological studies. We present an integrative framework combining GM, traditional machine learning (ML) algorithms, and convolutional neural networks (CNNs) for species discrimination. This study focused on the classification of digital wing images of *Aedes vittatus* and *Aedes aegypti* using a small binary dataset as a case study, and proposes a hybrid methodology integrating these approaches within a GM-based workflow. We evaluated multiple ML classifiers trained on principal component (PCA)-reduced features derived from GM. Furthermore, Procrustes-aligned and raw landmark coordinates were converted into rasterized grayscale images, enabling the application of CNNs to landmark-based shape data derived from GM. All approaches successfully discriminated the two *Aedes* species. During model development, traditional ML classifiers based on selected principal components achieved high internal performance, with the support vector machine (SVM) producing the strongest results among them. In contrast, CNN performance varied depending on network architecture and input representation. Among the evaluated models, the Baseline CNN exhibited the greatest stability, demonstrating consistent performance across input types and better generalization on independent data. Based on bootstrapped external validation, it achieved the highest overall classification performance, exceeding that of the best-performing ML model (SVM). The Deep CNN showed strong dependence on input representation, achieving acceptable performance only when trained on raw coordinate images that retained original size, orientation, and scale information. This highlights the sensitivity of deeper architectures to feature representation, particularly after Procrustes normalization. Notably, transfer learning using the pre-trained MobileNetV2 model performed poorly across all data formats, likely due to substantial domain mismatch between ImageNet natural images and the sparse, abstract representations of landmark-based GM data. Overall, this study establishes a proof-of-concept framework that integrates GM, ML, and CNN approaches, demonstrating their complementary strengths for species discrimination under data-limited conditions.

## Zusammenfassung

Die geometrische Morphometrie (Geometric Morphometrics, GM) ist ein etabliertes und kosteneffizientes Verfahren zur Quantifizierung von Formvariationen und wurde in den vergangenen Jahrzehnten umfassend eingesetzt, um morphologische Differenzierungen aufzuklären und nahe verwandte Taxa voneinander zu unterscheiden. Ein wesentlicher analytischer Vorteil der GM liegt in ihrem strukturierten Arbeitsablauf, durch den komplexe morphologische Merkmale in multivariate Formdaten überführt und gleichzeitig die Dimensionalität der Daten effizient berücksichtigt werden kann. Folglich stellen GM-Ergebnisse leistungsfähige, optimierte Merkmalsdatensätze für das Training von Klassifikationsmodellen des maschinellen Lernens in taxonomischen und ökologischen Studien dar. Wir präsentieren einen integrativen Ansatz, der GM, traditionelle Algorithmen des maschinellen Lernens (ML) und Convolutional Neural Networks (CNNs) zur Unterscheidung von Arten kombiniert. Diese Studie konzentrierte sich auf die Klassifikation digitaler Flügelbilder von Aedes vittatus und Aedes aegypti anhand eines kleinen binären Datensatzes als Fallstudie und schlägt eine hybride Methodik vor, die diese Ansätze innerhalb eines GM-basierten Arbeitsablaufs integriert. Wir evaluierten mehrere ML-Klassifikatoren, die mit durch die Hauptkomponentenanalyse (PCA) reduzierten Merkmalen trainiert wurden, die aus der GM abgeleitet waren. Darüber hinaus wurden Procrustes-ausgerichtete sowie rohe Landmarkenkoordinaten in gerasterte Graustufenbilder umgewandelt, wodurch die Anwendung von CNNs auf aus GM abgeleitete, landmarkenbasierte Formdaten ermöglicht wurde. Alle Ansätze waren erfolgreich bei der Unterscheidung der beiden Aedes-Arten. Während der Modellentwicklung zeigten traditionelle ML-Klassifikatoren auf Basis ausgewählter Hauptkomponenten eine hohe interne Leistungsfähigkeit, wobei die Support Vector Machine (SVM) unter diesen Klassifikatoren die besten Ergebnisse erzielte. Im Gegensatz dazu variierte die Leistung der CNNs in Abhängigkeit von der Netzwerkarchitektur und der Darstellung der Eingabedaten. Unter den untersuchten Modellen zeigte das Baseline-CNN die höchste Stabilität, mit einer konsistenten Leistung über verschiedene Eingabetypen hinweg und einer besseren Generalisierungsfähigkeit gegenüber unabhängigen Daten. Auf Grundlage der externen Validierung mittels Bootstrap-Verfahren erzielte es die insgesamt höchste Klassifikationsleistung und übertraf damit das leistungsstärkste ML-Modell (SVM). Das Deep-CNN zeigte eine starke Abhängigkeit von der Darstellung der Eingabedaten und erreichte eine akzeptable Leistung nur dann, wenn es mit Rohkoordinatenbildern trainiert wurde, die die ursprünglichen Informationen zu Größe, Orientierung und Maßstab beibehielten. Dies verdeutlicht die Sensitivität tieferer Architekturen gegenüber der Merkmalsrepräsentation, insbesondere nach der Procrustes-Normalisierung. Bemerkenswerterweise zeigte das Transferlernen mit dem vortrainierten MobileNetV2-Modell über alle Datenformate hinweg eine schwache Leistung, was wahrscheinlich auf eine erhebliche Domänenabweichung zwischen den natürlichen Bildern von ImageNet und den spärlichen, abstrakten Repräsentationen landmarkenbasierter GM-Daten zurückzuführen ist. Insgesamt etabliert diese Studie einen Proof-of-Concept-Ansatz, der GM-, ML- und CNN-Verfahren integriert und ihre komplementären Stärken für die Artunterscheidung unter Bedingungen begrenzter Datenverfügbarkeit demonstriert.

## Abstrak

Morfometri geometri (Geometric Morphometrics, GM) merupakan pendekatan yang telah mantap dan menjimatkan kos untuk mengkuantifikasi variasi bentuk, serta telah digunakan secara meluas sejak beberapa dekad yang lalu untuk menjelaskan pembezaan morfologi dan membezakan antara takson yang berkait rapat. Salah satu kelebihan analisis utama GM terletak pada aliran kerjanya yang tersusun, yang menukarkan ciri morfologi kompleks kepada data bentuk multivariat sambil mengurus dimensi data secara berkesan. Oleh itu, output GM berfungsi sebagai set ciri yang berkuasa dan dioptimumkan untuk melatih pengelas pembelajaran mesin dalam kajian taksonomi dan ekologi. Kami membentangkan satu rangka kerja integratif yang menggabungkan GM, algoritma pembelajaran mesin (ML) tradisional, dan rangkaian neural konvolusi (CNN) untuk pembezaan spesies. Kajian ini memberi tumpuan kepada pengelasan imej sayap digital Aedes vittatus dan Aedes aegypti menggunakan set data binari bersaiz kecil sebagai kajian kes, serta mencadangkan metodologi hibrid yang mengintegrasikan pendekatan-pendekatan ini dalam aliran kerja berasaskan GM. Kami menilai beberapa pengelas ML yang dilatih menggunakan ciri yang dikurangkan melalui analisis komponen utama (PCA) yang diperoleh daripada GM. Selain itu, koordinat tanda aras yang diselaraskan menggunakan Procrustes dan koordinat tanda aras mentah ditukarkan kepada imej skala kelabu yang dirasterkan, membolehkan CNN diaplikasikan kepada data bentuk berasaskan tanda aras yang diperoleh daripada GM. Semua pendekatan berjaya membezakan kedua-dua spesies Aedes. Semasa pembangunan model, pengelas ML tradisional berasaskan komponen utama terpilih menunjukkan prestasi dalaman yang tinggi, dengan mesin vektor sokongan (SVM) menghasilkan keputusan terbaik dalam kalangan pengelas tersebut. Sebaliknya, prestasi CNN berbeza bergantung pada seni bina rangkaian dan representasi input. Antara model yang dinilai, Baseline CNN menunjukkan kestabilan tertinggi, dengan prestasi yang konsisten merentas jenis input serta keupayaan generalisasi yang lebih baik terhadap data bebas. Berdasarkan pengesahan luaran menggunakan kaedah bootstrap, model tersebut mencapai prestasi pengelasan keseluruhan tertinggi, mengatasi model ML yang berprestasi terbaik (SVM). Deep CNN menunjukkan kebergantungan yang kuat terhadap representasi input, dengan prestasi yang boleh diterima hanya apabila dilatih menggunakan imej koordinat mentah yang mengekalkan maklumat asal mengenai saiz, orientasi dan skala. Dapatan ini menunjukkan kepekaan seni bina yang lebih mendalam terhadap representasi ciri, khususnya selepas normalisasi Procrustes. Menariknya, pembelajaran pemindahan menggunakan model MobileNetV2 yang telah dipra-latih menunjukkan prestasi yang lemah merentas semua format data, berkemungkinan disebabkan oleh ketidakpadanan domain yang ketara antara imej semula jadi ImageNet dengan representasi yang jarang dan abstrak bagi data GM berasaskan tanda aras. Secara keseluruhannya, kajian ini membangunkan satu rangka kerja bukti konsep yang mengintegrasikan pendekatan GM, ML dan CNN, serta menunjukkan kekuatan pelengkapnya untuk pembezaan spesies dalam keadaan data yang terhad.

## Introduction

Geometric morphometrics is a cost-effective approach for analyzing shape variation and has been widely used

for over three decades in taxonomic discrimination, including the identification of sibling and biologically cryptic species (Dujardin and Slice 2007; Combey et al. 2018; Deku et al. 2022). Beyond taxonomy, GM has also been applied to detect hybrids, identify invasive species, and investigate phenotypic divergence, making it a valuable tool in evolutionary and ecological research (Markovic et al. 2024; Hernandez-Martelo et al. 2026). Its relatively low cost ensures continued utility in resource-limited settings where molecular approaches remain less accessible. Moreover, studies integrating GM with molecular methods have frequently reported concordant findings, demonstrating that GM can provide reliable morphological evidence alongside genetic data (Geiger et al. 2016; Pérez‐Quiñónez et al. 2017; Sauer et al. 2020; Henriques et al. 2020).

Despite its successes, the efficacy of GM can be inconsistent in some cases, particularly when the taxonomic signal is not strongly shape-driven or when intraspecific shape variation within populations exceeds variation between them. This may arise when ecological influences, developmental plasticity, or high levels of phenotypic polymorphism obscure species-specific shape differences, thereby reducing the discriminatory power of GM classifiers. In addition, the linear statistical approaches commonly employed in GM studies, including Principal Component Analysis (PCA), Canonical Variate Analysis (CVA), and Discriminant Function Analysis (DFA), may not fully capture complex, non-linear patterns embedded within high-dimensional morphometric data under certain conditions (Du 2019; Wani 2025). This limitation is evident in microgeographical studies of Aedes aegypti, where GM has shown limited capacity to differentiate populations from distinct locations, likely due to high levels of within-population morphological variation (Henry et al. 2010; Vidal and Suesdek 2012; Hounkanrin et al. 2023). For example, in Benin, West Africa, GM failed to accurately classify populations despite clear genetic differentiation among locations (Hounkanrin et al. 2023). Similarly, Vidal and Suesdek (2012) demonstrated that GM could not reliably discriminate *Ae. aegypti* populations according to geographic origin, despite evidence of population structure based on microsatellite markers. Comparable challenges have been reported in other biological classification contexts, where morphological traits may exhibit limited resolution for identifying groups shaped by complex evolutionary processes (Courtenay et al. 2019; Bellin et al. 2021). Collectively, these findings highlight that the discriminatory performance of GM-based classifiers can be constrained under specific biological and ecological conditions.

Recent advances in computer vision and artificial intelligence have introduced ML and deep learning (DL) approaches as effective alternatives for biological classification, offering new opportunities to overcome limitations associated with conventional morphometric approaches (Ong and Suhalia Hamid 2022). In many applications, ML models successfully trained on morphometric-derived features from GM data enhance classification performance (Lorenz et al. 2015; Courtenay et al. 2019; Kasinathan et al. 2021; Belin et al. 2021; Salifu et al. 2022; Carmo et al. 2024). Deep learning models particularly, CNNs provide a distinct paradigm by enabling automated classification directly from raw images reducing cost, time and labor associated with conventional methods (Krizhevsky et al. 2012; Zhu et al. 2017; Motta et al. 2019; Park et al. 2020; Ong and Suhalia Hamid 2022). Transfer learning with state-of-the-art CNN architectures has become a valuable approach in taxonomy, especially under limited data conditions (Motta et al. 2019; Park et al. 2020). However, despite these advances, most existing studies either apply ML exclusively to predefined GM features or employ DL independently without direct integration or comparison with GM-derived representations. Consequently, systematic evaluations of traditional ML including DL approaches within a unified GM framework, particularly under data-limited conditions, remain scarce. The present study aims to bridge this gap by proposing an integrated comparative framework that combines GM with both traditional ML and CNN approaches. Specifically, the modelling framework comprised two parametric methods (Naïve Bayes [NB] and Logistic Regression [LR]), two non-parametric methods (Support Vector Machine [SVM] and Random Forest [RF]), and two ensemble learners (XGBoost and Soft Voting), as well as three CNN architectures: a baseline CNN, a deep CNN, and a transfer learning model (MobileNetV2). Collectively, this work provides a methodological baseline and proof of concept for integrating GM with ML and DL techniques for taxonomic classification and enhanced predictive performance under data-limited conditions.

## Materials and method

### Geometric morphometric

**Fig. 1:**
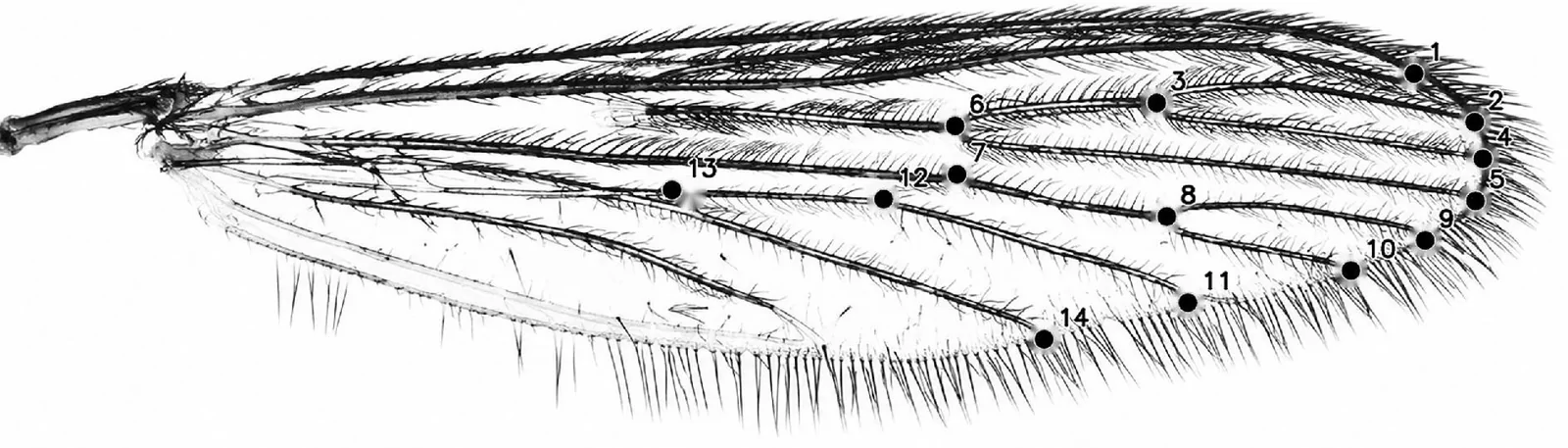
Landmark (L) configuration plotted on the right wing of an *Aedes* specimen: L1, distal end of the radial vein (R); L2, distal end of radial branch R2; L3, origin (fork) of radial branches R2 and R3; L4, distal end of radial branch R3; L5, distal end of radial branches R4 + R5; L6, radio-medial cross vein (r–m); L7, midpoint of medial vein branch M2; L8, radio-sectoral vein (Rs); L9, distal end of medial veins M1 + M2; L10, distal end of medial veins M3 + M4; L11, distal end of cubital vein Cu1; L12, midpoint of cubital vein Cu3; L13, origin of cubital vein Cu1; L14, distal end of cubital vein Cu2.

We applied landmark-based GM (LGM) to 254 female specimens from two *Aedes* species (*Aedes aegypti* [n = 138] and *Ae. vittatus* [n = 116]) based on venation patterns of the right forewing. The *Aedes* mosquito wing specimens used in this study were obtained from a previously published field survey conducted in Ghana, during the 2024 dengue outbreak (Deku et al. 2026). Voucher specimens are housed in the Insect Museum, University of Cape Coast, Ghana. Each specimen image was assigned a unique identifier and saved accordingly. A total of 14 landmark coordinates (Fig. 1), were selected corresponding to 14 morphological markers out of 18 markers used by Sendydiego et al. (2013) and other previous studies (Henry et al. 2010; Vidal and Suesdek 2012; Sauer et al. 2020). The markers are known taxonomic wing features used as reference points to describe wing patterns in both *Aedes* species. The remaining four landmarks were excluded because they showed inconsistent visibility and lower repeatability across specimens, which could introduce measurement error or unnecessary noise.

Digitization of landmark coordinates was performed using TPSDig2 (v.2.31), a commonly used tool in GM for generating Cartesian coordinates (Rohlf 2017). As part of quality control, established standardized protocols previously applied in GM analyses of these taxa were followed. Repeated digitization was performed during the initial landmarking process to ensure consistency and standardization in landmark placement. However, intra-observer measurement error was not formally quantified. Digitized data were imported into MorphoJ (Version 1.06d) for analysis (Klingenberg 2011), and two grouping variables were assigned: species identity (*Ae. aegypti* and *Ae. vittatus*) and dataset type (training and test sets). For each species, specimens were partitioned into training (70%) and test (30%) subsets. Specimens were assigned to training and test datasets using stratified simple random sampling, ensuring equal representation of both species. The same partitioning scheme was consistently applied across all subsequent ML and CNN analyses to ensure comparability of results. Randomization was performed using a fixed seed to ensure reproducibility, while the training and test datasets remained mutually exclusive throughout all analyses. However, GM analyses were performed using the full dataset, as the objective was to characterize species-specific shape variation rather than evaluate predictive performance. A Generalized Procrustes Analysis (GPA) was applied to align configurations and obtain shape variables free of size, position, and orientation effects, allowing comparative analyses based on Procrustes coordinates (Bookstein 1991). Shape variation was described as deviations from the mean landmark configuration. To distinguish species based on wing shape, we applied a multivariate statistical pipeline. Principal component analysis was first performed on the covariance matrix of Procrustes coordinates for dimensionality reduction and visualization of group separation in morpho-space (Mikery et al. 2019). The statistical significance of interspecific shape variation was then tested using Procrustes ANOVA (permutation multivariate analysis of variance on Procrustes coordinates) with 10,000 permutations to determine whether differences exceeded random variation. To quantify shape divergence, Procrustes and Mahalanobis distances between species means were calculated. The null hypothesis of no shape difference was assessed using 10,000 randomization iterations (Mikery et al. 2019; Sauer et al. 2020). Classification performance was evaluated using DFA, based on leave-one-out cross-validation (LOOCV) and summarized in a confusion matrix. To assess allometry, we performed a multivariate regression of shape on centroid size. In this model, Procrustes-aligned shape coordinates (represented by principal component scores) were treated as dependent variables, and log-transformed centroid size as the independent variable (Mikery et al. 2019). Centroid size was analyzed using IBM SPSS Statistics v26 (IBM Corp., Armonk, NY, USA) with a significance threshold of α = 0.05. Differences in centroid size between *Ae. aegypti* and *Ae. vittatus* were assessed using the Mann–Whitney U test.

### Preparation of morphometric datasets for ML and CNN analyses

**Fig. 2:**
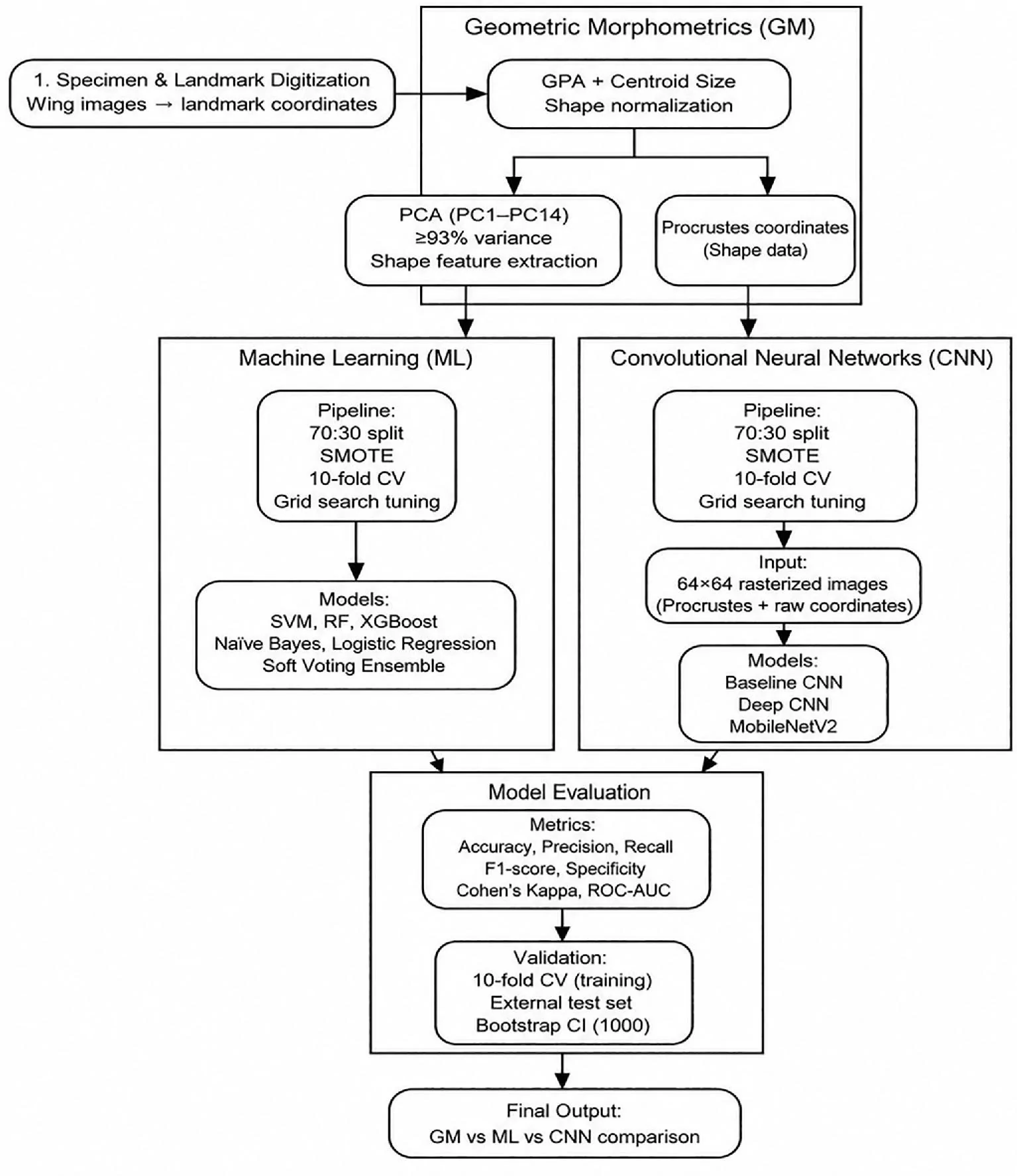
Workflow illustrating the integration of GM processing and ML/CNN classification. The pipeline begins with landmark digitization of mosquito wing samples followed by GPA for shape standardization. Three feature representations were generated from GM: raw landmark coordinates, Procrustes coordinates, and PCA-derived features. The PCA was used as input feature for traditional ML analysis while the landmark configurations (Procrustes coordinates, and raw landmark coordinates) are rasterized into RGB image format as input for the CNN-based analysis. Internal validation was performed using stratified 10-fold cross-validation on the training data. Final model performance was evaluated on an independent external test set.

The traditional ML models were trained on a subset of principal components (PCs) derived from geometric morphometric data, which captured the major patterns of shape variation between *Ae. aegypti* and *Ae. vittatus* and collectively explained a substantial proportion of total Procrustes shape variation. In contrast, the CNN models were trained directly on rasterized image representations generated from landmark coordinate data. Two CNN input types were evaluated: (i) images generated from Procrustes-aligned coordinates, representing shape information only, and (ii) images generated from raw landmark coordinates, which retained both shape- and size-related variation. Thus, the ML models operated in a PCA-reduced feature space, whereas the CNN models learned directly from image-based representations of morphometric coordinates (Fig. 2).

The ML and CNN models were trained separately using the training dataset and evaluated on an independent hold-out test dataset, which was randomly assigned during the geometric morphometric workflow. For the ML analyses, GPA was performed on the full dataset to obtain Procrustes-aligned coordinates. Principal component analysis was then fitted exclusively on the training data, and the resulting PCA transformation was used to project both training and test specimens into a common feature space. Consequently, the test specimens were projected onto PCA axes derived from the training set rather than being subjected to an independent PCA. This ensured that both datasets were represented within the same coordinate system and avoided inconsistencies arising from separate covariance structures. This approach also prevented information leakage between training and test sets during model development and evaluation. Although this approach differs from some strictly separable machine learning pipelines, it is widely accepted in geometric morphometrics, where PCA scores are commonly used as biologically interpretable summaries of shape variation rather than purely predictive features. This workflow is consistent with established GM and ML practices (Bellin et al. 2021; Burroughs et al. 2024).

For the CNN analyses, the training and test datasets were processed independently following dataset partitioning. However, GPA was performed separately within each dataset prior to image generation. Notably, the preprocessing strategies differed because the two analytical frameworks have different methodological requirements. For the ML pipeline, GPA was performed on the complete landmark dataset prior to PCA, consistent with standard geometric morphometric practice, as GPA itself is an unsupervised shape superimposition procedure that does not use class labels. In contrast, for the CNN pipeline, GPA was performed separately within the training and external test datasets before image generation so that all normalization parameters used for image construction were derived exclusively from the corresponding dataset, thereby maintaining a leakage-free preprocessing workflow. Preliminary comparisons indicated no discernible difference in model performance when GPA was performed separately or on the combined dataset. Nevertheless, the separate preprocessing strategy was retained because, unlike the PCA-based ML pipeline where the test data can be projected using the PCA model fitted on the training data, CNN image generation requires preprocessing to be completed prior to model training and evaluation.

### Traditional machine learning model development

For binary classification of the two *Aedes* mosquito species, six ML algorithms were trained and evaluated using shape variables (PCs) derived from GM data. The predictor variables consisted of the first fourteen principal components (PC1–PC14) out of 24 PCs, which collectively explained ≥ 93% of the total Procrustes shape variance between *Ae. aegypti* and *Ae. vittatus*. The machine learning (ML) classifiers were trained on data balanced using the Synthetic Minority Over-sampling Technique (SMOTE). Although the class imbalance was relatively modest, preliminary model development demonstrated consistently improved classification performance with SMOTE compared with models trained without oversampling or with class weighting alone. Accordingly, SMOTE was retained to provide balanced class representation during training. Oversampling was applied exclusively to the training dataset and model performance was evaluated using stratified 10-fold cross-validation restricted to the training set. The independent test dataset remained untouched throughout model development to ensure an unbiased assessment of model performance. All analyses were performed in Python 3.12.3 using the scikit-learn library, with XGBoost models implemented using the XGBoost library.

The modelling framework comprised two parametric approaches (Naïve Bayes [NB] and Logistic Regression [LR]), two non-parametric methods (Support Vector Machine [SVM] and Random Forest [RF]), and two ensemble learners (Extreme Gradient Boosting [XGBoost] and Ensemble Soft Voting).

Prior to model fitting, all predictors were standardized to zero mean and unit variance. Hyperparameters were optimized using grid search with cross-validation on the training data to maximize predictive performance and reduce overfitting. Models were implemented using the scikit-learn library. For RF, the number of trees (100 and 200) and maximum tree depth (None and 10) were evaluated, with the optimal model using 200 trees; all other parameters were retained at their default settings (criterion = “gini,” min_samples_split = 2, min_samples_leaf = 1, and max_features = “sqrt”). SVM used a radial basis function kernel, with hyperparameters C (1 and 10) and gamma (“scale” and “auto”) optimized, yielding an optimal model with C = 1 and gamma = “scale.” LR employed the LBFGS optimizer with L2 regularization, and regularization strengths (C = 0.1, 1, and 10) were evaluated, with C = 10 selected. For XGBoost, the number of estimators (100 and 200) and learning rates (0.05 and 0.1) were optimized, with the final model using 200 estimators and a learning rate of 0.1. NB used default parameters. Ensemble Soft Voting combined the class probabilities from all classifiers, with the final prediction assigned to the class with the highest averaged probability.

### Convolutional neural networks (CNN)

In our study, the CNN models were trained on raw images generated from raw coordinate data (landmarks with position, rotation and scale variation) and Procrustes coordinates (pure shape information excluding orientation or size related data) from GM contrasting the ML models which received training using the PCA scores. The coordinate datasets (normalized Procrustes-aligned and non-normalized raw coordinates) were processed using the Keras deep learning library to generate image representations for CNN analysis. Following stratified partitioning into training and external test datasets, the minimum and maximum x- and y-coordinate values were estimated exclusively from the training dataset and subsequently applied unchanged to normalize both datasets, thereby preventing information leakage. The normalized landmark coordinates were then rasterized onto a 64 × 64 pixel grid, with each homologous landmark represented as a single white pixel (pixel intensity = 255) on a black background (pixel intensity = 0), producing binary grayscale images that preserved the geometric configuration of the wing landmarks. The grayscale images were resized to 96 × 96 pixels using bilinear interpolation and converted to three-channel RGB format by replicating the grayscale channel to meet the input requirements of the CNN architectures, including MobileNetV2. The same image-generation workflow was applied independently to both the normalized Procrustes-aligned and non-normalized raw coordinate datasets, and species class labels were assigned before binary classification.

### Description of the CNN model architecture

Three convolutional neural network (CNN) architectures were implemented: a baseline CNN, a deeper CNN, and a transfer learning model (MobileNetV2). The models were developed using TensorFlow and Keras (tf.keras) and trained and evaluated on both Procrustes-derived and raw coordinate RGB image representations to compare performance across different feature encodings. All were trained on NVIDIA Tesla GPUs. The Baseline CNN is a simple model consisting of a simple convolutional and max pooling layer with high computational efficiency (Shin et al. 2016). The Baseline CNN served as a shallow, proof-of-concept architecture (Table S1) to assess the feasibility of using raw images for classification before employing more complex models. It accepts RGB-aligned wing images and comprised three convolutional layers for feature extraction. The first two layers used 32 and 64 filters (3 × 3), respectively, each followed by ReLU activation and 2 × 2 max-pooling. A third convolutional layer (64 filters, 3 × 3, ReLU) extracted higher-level features without pooling. The resulting feature maps were flattened and connected to a dense layer (64 units, ReLU) before a final sigmoid output unit for binary prediction. The model was compiled with the Adam optimizer and binary cross-entropy loss. The Customized Deep CNN architecture (Table S2) processed RGB-aligned wing images and extended the Baseline CNN with deeper feature extraction and richer dense representations. The network consisted of four convolutional blocks (3 × 3 convolution, ReLU activation, batch normalization, and 2 × 2 max-pooling), with filters increasing from 32 to 128. Feature maps were flattened and passed through two dense layers (128 and 64 units) with ReLU activation and 30% dropout, followed by a sigmoid output layer for binary classification. The model was compiled using the Adam optimizer with binary cross-entropy loss. Pooling layers reduce spatial dimensionality while retaining salient features, improving computational efficiency and robustness to noise (Kanani and Padole 2019; Hartbauer 2024). Fully connected layers integrate extracted features to produce final predictions, while ReLU introduces non-linearity to enable learning of complex patterns in image data. MobileNetV2 is a lightweight convolutional neural network architecture developed by Google and pretrained on approximately 1.4 million ImageNet images across 1,000 classes (Sandler et al. 2018). It is optimized for efficient performance in mobile and resource-constrained environments while maintaining strong classification accuracy (Hussain et al. 2022).

### Optimization and strategies to minimize model overfitting and bias

Each model was optimized through hyperparameter tuning using Keras Tuner (Random Search) to identify optimal architectural configurations and learning rates based on validation accuracy (Schratz et al. 2019; Ong and Suhalia Hamid 2022). Optimizers and learning rates were selected from a predefined search space. The final models were trained using SMOTE-balanced datasets and evaluated using 10-fold cross-validation to mitigate overfitting and reduce bias associated with limited sample size and class imbalance (Lachenbruch and Mickey 1968; Bellin et al. 2021; Adi Pratama and Oktora 2023; Baddah et al. 2024). Additionally, model complexity was controlled by adjusting the number of training epochs and applying a dropout rate of 0.3, selected after monitoring training and validation loss curves. Early stopping was also implemented to prevent over-training, while learning rates (0.001 and 0.0001) were systematically tested to identify optimal convergence behavior (Ong and Suhalia Hamid 2022). Dropout has been widely recognized as an effective regularization technique, particularly in deep learning applications involving limited datasets (Brigato and Iocchi 2021). Final optimized models were subsequently evaluated on an independent external test set that was not used during training or tuning.

### Model performance assessment

The performance of the GM, ML, and CNN models was evaluated using accuracy, precision, recall (sensitivity), specificity, F1-score, area under the receiver operating characteristic curve (AUC-ROC), and Cohen’s kappa. Accuracy, precision, recall, specificity, F1-score, and Cohen’s kappa statistics were calculated from confusion matrices generated from the predicted and observed class labels (Ong and Suhalia Hamid 2022; Salifu et al. 2022). In contrast, ROC curves and AUC values were computed from predicted class probabilities across varying classification thresholds (Jiménez-Valverde 2012). Model performance during development was assessed using stratified 10-fold cross-validation on the training dataset to evaluate internal robustness. External validation of the ML and CNN models was conducted using the independent test dataset, with uncertainty in performance estimates quantified through bootstrapping (1,000 resamples with replacement) to generate 95% confidence intervals for each metric (Efron 1992).

## Results

### Geometric morphometric

Unlike ML and CNN approaches, GM analyses were conducted on full datasets to evaluate the reproducibility of species-specific shape patterns rather than predictive generalization. Principal component analysis (PCA) revealed that the first three principal components accounted for 52.0% of total Procrustes shape variance (PC1: 26.5%, PC2: 15.8%, PC3: 9.6%), and visualization of the resulting morpho-space showed separation between Ae. aegypti and Ae. vittatus. The first sixteen principal components (PCs), each with an eigenvalue greater than one, cumulatively explained 95.2% of the total shape variation. The morphological distinction between *Ae. aegypti* and *Ae. vittatus* was statistically robust, as confirmed by highly significant Procrustes distance (0.030) and Mahalanobis distance (2.62) values (*P* < 0.0001). Visualizations of shape change along the major PCs indicated that landmarks 3, 6, 7, 8, and 10–14 (particularly landmarks 8, 10, and 11) were the primary contributors to the observed species separation. Procrustes ANOVA was significant for shape (df = 24,5832, F = 6.55, *P* < 0.0001) and centroid size (df = 1,243 F = 61.75, *P* < 0.0001). Discriminant Function Analysis (DFA) indicate distinct morphological groups. Leave-one-out cross-validation (LOOCV) of the DFA yielded an overall classification accuracy of 80.7%. This differentiation was driven by consistent shape changes in specific venation landmarks. Critical morphological markers identified include deviations in the origin of radial branches 2 and 3 (landmark 3), the radio-sectoral vein, and the origin of the cubital vein 1, highlighting their importance in species-specific wing patterning. Centroid size analyses revealed differentiation. *Aedes vittatus* females: 526.1 (95%CI: 515.4–536.7) obtained significantly larger wingspan than *Ae. aegypti* females 431.8 (95%CI: 429.6–436.7) (Mann-Whitney U = 14,745; *P* < 0.0001). Multivariate regression revealed a significant allometric relationship between wing shape and log centroid size (*P* < 0.0001). Examination of the PCA morpho-space indicated that the allometric pattern was primarily aligned with variation along PC2. Regression of shape on log centroid size showed that the allometric vector was most strongly associated with PC2 (10.3%), whereas PC1 contributed only 0.35%, indicating that size-related shape variation was concentrated primarily along the second principal component. The remaining principal components exhibited little evidence of allometric association. Overall, centroid size contributed minimally to wing shape variation, indicating that allometric effects accounted for only a small proportion of the observed morphological differences.

### Traditional machine learning algorithms

Table 1 summarizes the internal performance metrics of all evaluated models. The traditional ML models trained on a subset of principal components (14 PCs) demonstrated high internal classification performance in discriminating between the two *Aedes* species. Mean cross-validation accuracies, precision, and specificity ranged from 82–90%, 83–96%, and 87–97%, respectively, across the six classifiers. Among these, the support vector machine (SVM) achieved the highest overall performance, yielding the highest accuracy (90%), precision (96%), F1 score (86%), and Cohen’s kappa (79%). In contrast, Naïve Bayes produced the lowest accuracy and F1 scores and exhibited significantly lower sensitivity comparatively.

**Table 1:** Cross validation metrics (95%CI) of GM, ML and CNN models using PCA-derived features

Model type	Input data	Specificity	Sensitivity (recall)	Precision	F1 score	Accuracy	Kappa
Geometric morphometrc (DFA/LDA)	Full PCs	0.84 (0.75– 0.90)	0.75 (0.65–0.85)	0.77 (0.68– 0.86)	0.76 (0.68– 0.84)	0.80 (0.74 –0.86)	0.59 (0.47– 0.71)
Support Vector Machine	14 PCs	0.97 (0.93–1.00)	0.81 (0.72–0.88)	0.96 (0.92–1.00)	0.8621 (0.79–0.93)	0.90 (0.86–0.95)	0.79 (0.72–0.87)
Logistic regression	14 PCs	0.87 (0.84–0.90)	0.82 (0.77– 0.88)	0.83 (0.78–0.88)	0.8179 (0.77–0.86)	0.85 (0.82– 0.88)	0.69 (0.64– 0.74)
Random forest	14 PCs	0.95 (0.92–0.98)	0.76 (0.68– 0.83)	0.92 (0.85– 0.98)	0.82 (0.75– 0.88)	0.87 (0.83– 0.91)	0.72 (0.64–0.81)
Naïve bayes	14 PCs	0.948 (0.90–0.98)	0.66 (0.58–0.73)	0.90 (0.83–0.97)	0.75 (0.69–0.81)	0.82 (0.79–0.86)	0.62 (0.56–0.68)
XGboost	14 PCs	0.90 (0.87–0.93)	0.79 (0.73–0.85)	0.85 (0.80–0.90)	0.8175 (0.76–0.87)	0.86 (0.82–0.89)	0.70 (0.64–0.76)
Ensemble soft voting	14 PCs	0.95 (0.93–0.98)	0.78 (0.73–0.83)	0.93 (0.89–0.97)	0.84 (0.80–0.89)	0.88 (0.85– 0.91)	0.75 (0.70–0.80)
Simple baseline CNN	Procrustes Coords	0.83 (0.61– 1.00)	0.77 (0.53– 0.98)	0.83 (0.69– 1.00)	0.79 (0.64–0.97)	0.80 (0.69–0.97)	0.61 (0.37– 0.93)
Deep CNN	Procrustes Coords	0.00 (0.00– 0.00)	1.00	0.50 (0.47– 0.53)	0.67(0.64– 0.68)	0.50 (0.47– 0.53)	0.00(0.00– 0.00)
MobileNetTV2 (Transfer learning)	Procrustes Coords	0.60 (0.00– 1.00)	0.40 (0.00–1.00)	0.20 (0.00– 0.52)	0.26 (0.00– 0.68)	0.50 (0.47– 0.52)	0.00 (0.00– 0.00)
Simple baseline CNN	Raw Coord.	0.74	0.75 (0.62– 0.88)	0.71 (0.64– 0.78)	0.73 (0.63–0.82)	0.72 (0.64– 0.81)	0.45 (0.28– 0.62)
Deep CNN	Raw Coord.	0.74	0.78 (0.68– 0.88)	0.77 (0.68– 0.87)	0.77(0.69–0.85)	0.77(0.68–0.85)	0.53(0.35– 0.71)
MobileNetTV2(Transfer learning)	Raw Coord.	0.74	0.00	0.30(0.16– 0.49)	0.40(0.15– 0.65)	0.51 (0.49– 0.52)	0.00

*Notes*: The GM (DFA/LDA) was evaluated on full PCA-derived features. The MLs were trained on partial PCA-derived features (14PCs). The CNN models were trained directly on rasterized image representations generated from landmark coordinate data (both Procrustes-aligned and raw coordinate data).

### Convolutional neural network models

Performance of the CNNs varied by inputs, the simple Baseline CNN showed the most consistent performance across both Procrustes-transformed and raw coordinate image inputs, achieving mean accuracies of approximately 72–80% with moderate sensitivity and precision (Table 1). In contrast, the customized Deep CNN exhibited strong dependence on input representation. When trained on images generated from Procrustes-transformed coordinates, performance was poor, with low specificity, precision, F1 score, and near-random agreement (κ ≈ 0). However, training on raw coordinate images resulted in marked improvements across all performance metrics, including sensitivity, precision, F1 score, and overall accuracy, with performance comparable to or exceeding that of the simple Baseline CNN trained on raw coordinate data (Table 1). These results indicate that the deeper architecture benefited from the richer spatial information retained in the raw coordinate representations and was better able to capture complex landmark configurations present in the original data. In contrast, Transfer learning using MobileNetV2 performed poorly irrespective of input representation. Models trained on both Procrustes and raw coordinate images exhibited low specificity, precision, F1 score, and Cohen’s kappa, with several metrics approaching or falling below chance-level classification. These findings suggest limited transferability of features learned from natural image datasets to sparse, binary, landmark-derived morphometric images.

### Comparative analyses between GM, ML and CNNs

Comparatively, the traditional ML models trained on a subset of PCA-derived features achieved the high internal classification performance during binary discrimination of the two *Aedes* species, with overall mean cross-validation accuracies (82% to 90%), generally exceeding the classification accuracy obtained using GM alone (80%) and the CNN models (51–80%) (Table 1). Most performance metrics, including specificity, F1 score, Cohen’s kappa, precision, and sensitivity (recall), were also higher for the ML models than for GM and CNN approaches. Notably, GM analysis and the best-performing CNN architecture (simple Baseline CNN trained on Procrustes images) exhibited comparable internal performance, although the CNN achieved slightly higher F1 score, precision, and sensitivity values. However, when trained on raw coordinate images that retained size-related and orientation information, the Baseline CNN exhibited reduced internal performance, with accuracy and precision declining to levels comparable to those achieved by GM and the Deep CNN (Table 1).

### External test evaluation of ML and CNN

**Fig. 3:**
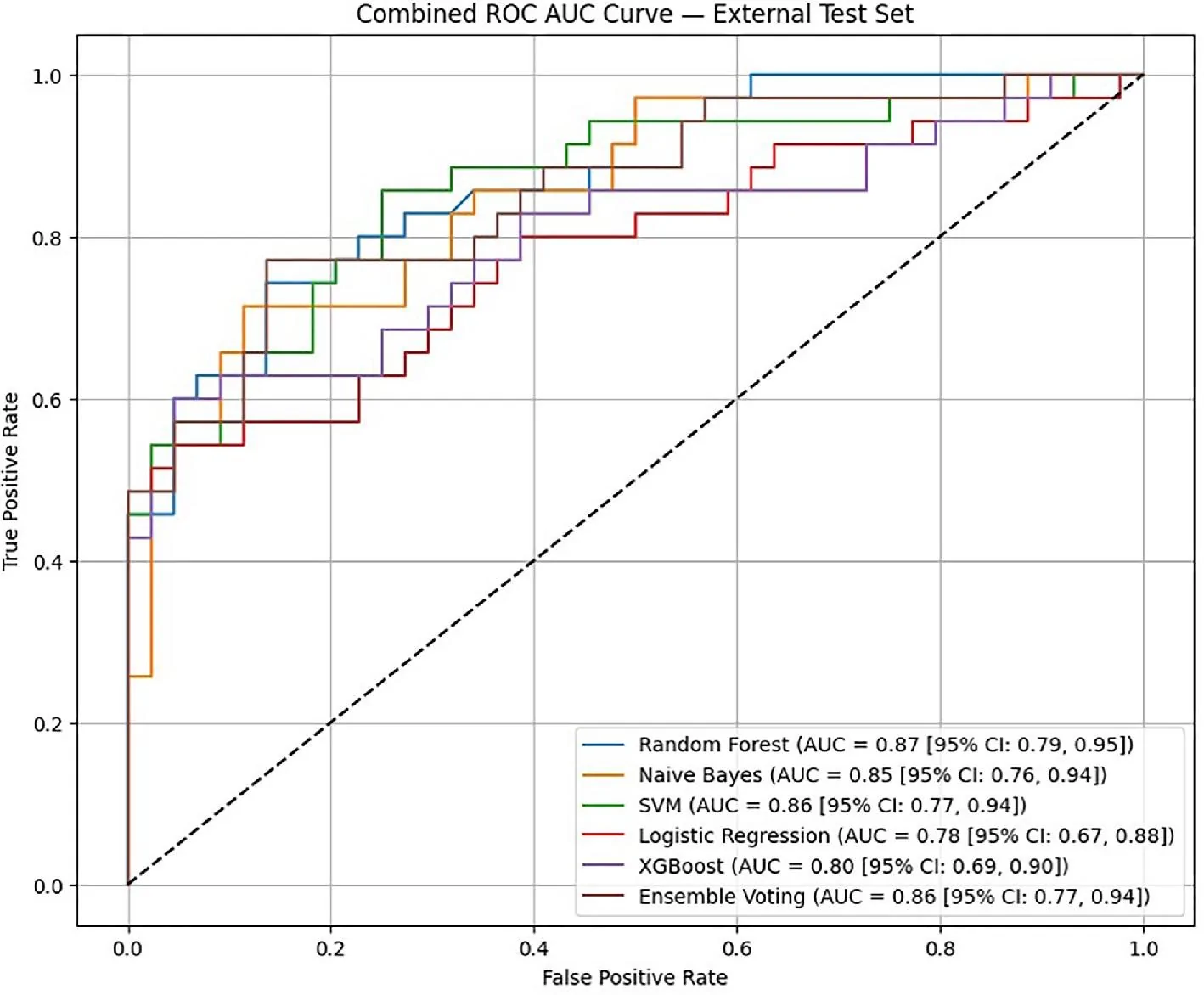
Combined receiver operating characteristic (ROC) curves of the machine learning (ML) models during external testing using principal component (PC) scores derived from geometric morphometric analysis. Model performance was evaluated using the area under the ROC curve (AUC), with higher AUC values indicating superior classification accuracy. The diagonal dashed line denotes the expected performance of a random classifier (AUC = 0.50).

**Fig. 4:**
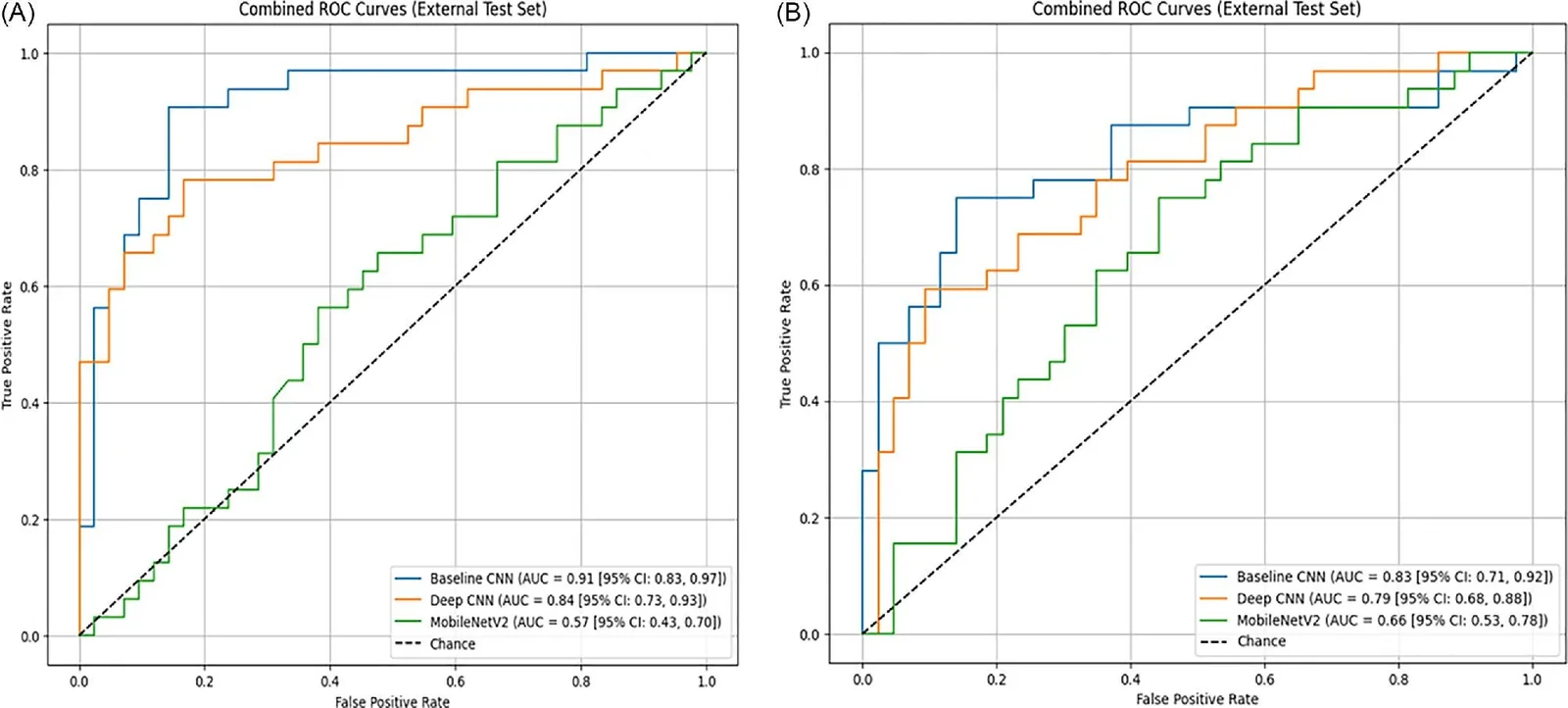
Combined receiver operating characteristic (ROC) curves of the convolutional neural network (CNN) models during the testing phase using Procrustes coordinate images (a) and raw coordinate images (b). The curves illustrate the classification performance of the Baseline CNN, Deep CNN, and MobileNetV2 models in distinguishing the two *Aedes* species. Higher AUC values indicate superior classification performance. The diagonal reference line represents random classification (AUC = 0.50).

During external validation, the performance of the traditional ML models declined relative to their internal cross-validation results, with reductions in accuracy ranging from approximately 6 to 13% (Table 2). Consequently, most external performance metrics indicated only moderate classification ability. In contrast, the simple Baseline CNN trained on Procrustes images demonstrated the strongest overall performance among all evaluated models and achieved bootstrapped estimates comparable to those obtained during training indicating high generalization potential (Table 2; Figs 3 and 4). The model achieved highest external accuracy (83%) and AUC (91%), while also exhibiting high Cohen’s kappa statistics, sensitivity, and F1 score relative to the other models (Fig. 4). When evaluated using raw coordinate images, the simple Baseline CNN produced moderate performance metrics that were broadly consistent with those observed during training and achieved classification performance comparable to that of the ML models (Table 2). These findings suggest that the Baseline CNN maintained relatively stable performance across input representations. A similar pattern was observed for the customized Deep CNN trained on raw coordinate images, which maintained moderate performance during both internal and external evaluation. When trained on Procrustes image inputs, external test results mirrored the trends observed during training, with the Deep CNN exhibiting consistently poor classification performance contrasting the raw coordinate results (Table 2; Fig. 4). The transfer learning model MobileNetV2 consistently showed the lowest internal and external performance regardless of input representation indicating limited transferability of features learned from natural image datasets to landmark-derived morphometric images (Table 2; Fig. 3).

**Table 2:** Bootstrapped evaluation metrics (95% CI) for the external test performance of ML and CNN models

Model type	Input data	Specificity	Sensitivity (Recall)	Precision	F1 score	Accuracy	Kappa
SVM	14 PCs	0.93 (0.84– 1.00)	0.54 (0.39– 0.71)	0.86 (0.70– 1.00)	0.67 (0.52– 0.80)	0.76 (0.67– 0.84)	0.49 (0.31– 0.68)
Logistic regression	14 PCs	0.8409 (0.74– 0.94)	0.57(0.41– 0.74)	0.74 (0.57– 0.88)	0.65 (0.50– 0.77)	0.72 (0.63– 0.82)	0.42(0.23– 0.61)
Random forest	14 PCs	0.95 (0.88– 1.00)	0.51(0.35– 0.68)	0.90 (0.74– 1.00)	0.65 (0.49– 0.79	0.75 (0.67– 0.86)	0.49(0.31– 0.68)
Naïve bayes-BTP	14 PCs	0.93 (0.85– 1.00)	0.57 (0.42– 0.74)	0.87 (0.71– 1.00)	0.69 (0.55– 0.81)	0.77 (0.68– 0.86)	0.52(0.35– 0.70)
XGboost	14 PCs	0.95(0.88– 1.00)	0.57 (0.41– 0.74)	0.91 (0.76– 1.00)	0.70 (0.55– 0.83)	0.78 (0.70– 0.87)	0.55(0.37– 0.73)
Ensemble Voting	14 PCs	0.93(0.84– 1.00)	0.57(0.42– 0.74)	0.87 (0.71– 1.00)	0.69 (0.55– 0.81)	0.77 (0.68– 0.86)	0.52(0.33– 0.71)
Simple baseline CNN	Procrustes Coords	0.89 (0.78– 0.98)	0.75 (0.59– 0.90)	0.83(0.68– 0.96)	0.79 (0.66– 0.89)	0.83 (0.75– 0.90)	0.64(0.47– 0.81)
Deep CNN	Procrustes Coords	0.00 (0.00–0.00)	1.00 (1.00–1.00)	0.43 (0.32–0.53)	0.60(0.48–0.70)	0.43 (0.32–0.53)	0.00 (0.00–0.00)
MobileNetV2	Procrustes Coords	0.00	1.00	0.43(0.32–0.55)	0.60(0.48 0.71)	0.43 (0.32–0.54)	0.00
Simple baseline CNN-BTP	Raw Coord.	0.71 (0.71–0.72)	0.84 (0.84–0.85)	0.69 (0.69–0.70)	0.76(0.75–0.76)	0.77(0.767–0.77)	0.54 (0.53–0.55)
Deep CNN-BTP	Raw Coord.	0.66(0.66–0.67)	0.91(0.90–0.91)	0.67(0.67–0.68)	0.77(0.76–78)	0.77(0.765–0.77)	0.55 (0.54–0.55)
MobileNetV2	Raw Coord.	1.00	0.00	0.00	0.00	0.56(0.56–0.57)	0.00

## Discussion

The integration of GM with ML algorithms and CNN models established a methodological baseline and provided proof of concept for taxonomic classification under data-limited conditions. Preliminary GM analyses effectively discriminated between the two Aedes species, consistent with previous studies demonstrating the utility of GM for mosquito species discrimination (Geiger et al. 2016; Pérez-Quiñónez et al. 2017; Sauer et al. 2020). Application of ML models to PCA-derived morphometric features generally improved classification performance relative to conventional GM approaches, although differences among some models were modest. These findings agree with contemporary studies showing that combining GM with ML can perform better than traditional statistical classifiers (Lorenz et al. 2015; Courtenay et al. 2019; Kasinathan et al. 2021; Bellin et al. 2021; Salifu et al. 2022). The comparatively lower performance of Naïve Bayes in some metrics likely reflects its assumption of predictor independence, which may be unrealistic for correlated morphometric variables. In contrast, models such as SVM demonstrated robust performance during training supporting their integration into standard GM analytical pipelines consistent with previous studies (Bellin et al. 2021; Salifu et al. 2022). The success of the traditional ML models can largely be attributed to PCA, which reduces dimensionality while retaining the most informative components of wing shape variation, thereby providing cleaner feature spaces for classification. These findings are consistent with theoretical expectations that traditional ML approaches often perform better than DL models when datasets are small because of their lower complexity and reduced data requirements (Salifu et al. 2022). Convolutional neural networks trained on image representations derived from GM data also achieved promising classification performance, demonstrating their potential application within GM-based workflows. Although CNNs are primarily designed for raw image analysis, their application to landmark-derived morphometric images remains relatively uncommon. Among the evaluated architectures, the simple Baseline CNN showed the most consistent performance across both Procrustes-transformed and raw coordinate images. Notably, Procrustes-derived images yielded results broadly comparable to those obtained with traditional ML models. The high internal performance of the Baseline CNN likely reflects the ability of simpler architectures to capture discriminative shape information while avoiding over-parameterization under limited sample sizes. In contrast, the customized Deep CNN displayed greater dependence on input representation. Acceptable internal performance was achieved only with raw coordinate images, which retained additional size-, scale-, and orientation-related information. This suggests that deeper architectures may benefit from the richer spatial information present in original coordinates, whereas Procrustes transformation may remove features useful for complex representation learning. The reduced performance of the Deep CNN on Procrustes images is also consistent with overfitting and optimization difficulties commonly encountered when training deep networks with limited data (Brigato and Iocchi 2021). Similarly, transfer learning using MobileNetV2 consistently produced the lowest performance regardless of input representation, likely because features learned from natural images in ImageNet are poorly suited to sparse landmark-based morphometric images. Notably, for the Procrustes coordinates used in CNN training, we observed no appreciable differences in classification performance whether GPA was performed separately on the training and external test datasets or on the full dataset prior to partitioning. Thus, the choice of GPA preprocessing strategy did not materially influence CNN classification outcomes in the present study. Nevertheless, future studies should adopt fully harmonized, leakage-free preprocessing pipelines to facilitate direct methodological comparisons between machine learning and deep learning approaches.

External validation revealed a decline in performance of most traditional ML models compared with internal cross-validation results, possibly reflecting the limited sample sizes available for training and testing. In contrast, CNN models exhibited greater consistency between internal and external evaluations. In particular, the simple Baseline CNN maintained comparable performance across independent datasets, indicating better generalization than the traditional ML approaches. This robustness may partly stem from the use of full Procrustes coordinate information, which preserves more shape information than the reduced PCA features used for ML training. Collectively, these findings highlight the importance of balancing model complexity with data representation and suggest that relatively simple CNN architectures may provide a promising and reliable solution for small landmark-based morphometric datasets.

## Conclusion, limitations and recommendation

This proof-of-concept study demonstrates the feasibility of integrating GM, ML, and CNN (GM–ML–CNN) for taxonomic classification using mosquito wing landmarks. Traditional ML models trained on PCA-derived variables achieved the highest cross-validation performance, whereas the simple Baseline CNN showed greater stability during external validation. These findings suggest that GM, ML, and CNN approaches are complementary rather than replacements for one another. Under the relatively small sample size used, simpler CNN architectures performed comparably to or more consistently than deeper and transfer learning models. Several limitations should be considered. The modest sample size may have affected model complexity and performance estimates, and the generalizability of these models across populations’ remains uncertain. In addition, the biological interpretation of CNN-derived features is still limited, and no widely adopted framework currently exists for integrating GM with ML and DL approaches. Future studies should incorporate larger, geographically diverse datasets and standardized validation procedures to better assess the robustness and transferability of this integrative framework. Overall, the GM–ML–CNN approach provides a promising complementary strategy for taxonomic classification under limited data conditions.

## Supplementary Material

obag048_Supplemental_File

## Data Availability

The data will be made available upon request from the corresponding author.
