# Supplementary material for: Integrating Traditional Machine Learning and Convolutional Neural Networks Into the Geometric Morphometric Pipeline for Taxonomic Classification (GM-ML-CNN)
Source: Integr Org Biol. 2026 Aug 21;8(1):obag048. doi: 10.1093/iob/obag048 (PMC13531468; doi:10.1093/iob/obag048)
Supplement: Supplementary file 1 [file obag048_supplemental_file.docx]

*Table S1*. Summary of customized Baseline CNN model specifications

- Input: Procrustes-aligned RGB wing images.

**Feature Extraction Backbone:**

Conv Block 1: Conv2D (32 filters, 3×3) → ReLU → MaxPooling2D (2×2)

Conv Block 2: Conv2D (64 filters, 3×3) → ReLU → MaxPooling2D (2×2)

Conv Layer 3: Conv2D (64 filters, 3×3) → ReLU

Classifier Head: Flatten → Dense (64 units, ReLU) → Dense (1 unit, Sigmoid)

- Compilation: Optimizer = Adam; Loss = Binary Cross-Entropy.

*Table S2*. Summary of Customized Deep CNN model specifications

- Input: Procrustes-aligned RGB wing images.

**Feature Extraction (Four Sequential Convolutional Blocks):**

1. Conv2D (32 filters, 3×3, ReLU) → BatchNorm → MaxPooling2D (2×2)

1. Conv2D (64 filters, 3×3, ReLU) → BatchNorm → MaxPooling2D (2×2)

1. Conv2D (128 filters, 3×3, ReLU) → BatchNorm → MaxPooling2D (2×2)

1. Conv2D (128 filters, 3×3, ReLU) → BatchNorm → MaxPooling2D (2×2)

- Flattening: Converts 2D feature maps to a 1D vector.

**Fully Connected Classifier:**

- Dense (128 units, ReLU) → Dropout (0.3)

- Dense (64 units, ReLU) → Dropout (0.3)

- Dense (1 unit, Sigmoid activation)

- Compilation: Optimizer = Adam; Loss = Binary Cross-Entropy.
